# *trans*-Dioxidotetra­pyridine­rhenium(V) triiodide

**DOI:** 10.1107/S1600536809030724

**Published:** 2009-08-12

**Authors:** Miłosz Siczek, Marta S. Krawczyk, Tadeusz Lis

**Affiliations:** a University of Wrocław, Faculty of Chemistry, 14 Joliot-Curie St, 50-383 Wrocław, Poland

## Abstract

In the title salt, [ReO_2_(C_5_H_5_N)_4_]I_3_, the cation and anion are both located on centres of symmetry. The Re^V^ atom adopts a *trans*-ReO_2_N_4_ octa­hedral coordination and short intra­molecular C—H⋯O contacts occur within the cation. In the crystal, the cations form layers perpendicular to [100] and a weak C—H⋯O inter­action links the cations.

## Related literature

For related structures containing the same cation, see: Calvo *et al.* (1971 ▶); Lock & Turner (1978 ▶); Luck & O’Neill (2001 ▶). For further synthetic details, see: Johnson *et al.* (1967 ▶). For background to aromatic π–π stacking, see: Janiak (2000 ▶).
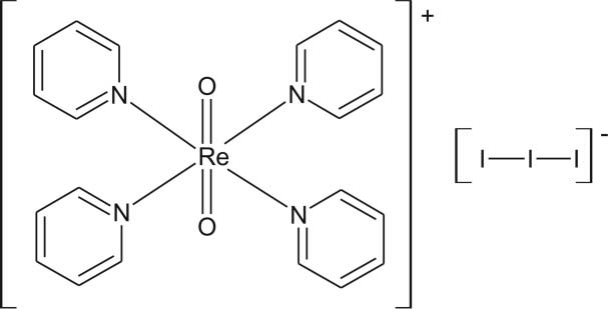


## Experimental

### 

#### Crystal data

[ReO_2_(C_5_H_5_N)_4_]I_3_
*M* *_r_* = 915.30
Triclinic, 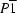

*a* = 7.993 (3) Å
*b* = 9.100 (3) Å
*c* = 9.356 (3) Å
α = 92.45 (4)°
β = 102.41 (4)°
γ = 104.10 (4)°
*V* = 641.3 (4) Å^3^
*Z* = 1
Mo *K*α radiation
μ = 8.37 mm^−1^
*T* = 100 K
0.10 × 0.10 × 0.07 mm

#### Data collection

Oxford Diffraction Xcalibur PX KM-4-CCD diffractometer
Absorption correction: analytical (*CrysAlis RED*; Oxford Diffraction, 2006 ▶) *T*_min_ = 0.411, *T*_max_ = 0.656
11244 measured reflections
4298 independent reflections
3593 reflections with *I* > 2σ(*I*)
*R*_int_ = 0.027

#### Refinement

*R*[*F* ^2^ > 2σ(*F* ^2^)] = 0.019
*wR*(*F* ^2^) = 0.031
*S* = 1.04
4298 reflections
139 parameters
H-atom parameters constrained
Δρ_max_ = 1.40 e Å^−3^
Δρ_min_ = −1.08 e Å^−3^

### 

Data collection: *CrysAlis CCD* (Oxford Diffraction, 2006 ▶); cell refinement: *CrysAlis RED* (Oxford Diffraction, 2006 ▶); data reduction: *CrysAlis RED*; program(s) used to solve structure: *SHELXS97* (Sheldrick, 2008 ▶); program(s) used to refine structure: *SHELXL97* (Sheldrick, 2008 ▶); molecular graphics: *XP* in *SHELXTL* (Sheldrick, 2008 ▶); software used to prepare material for publication: *SHELXL97* and *publCIF* (Westrip, 2009 ▶).

## Supplementary Material

Crystal structure: contains datablocks I, global. DOI: 10.1107/S1600536809030724/hb5027sup1.cif

Structure factors: contains datablocks I. DOI: 10.1107/S1600536809030724/hb5027Isup2.hkl

Additional supplementary materials:  crystallographic information; 3D view; checkCIF report

## Figures and Tables

**Table 1:** Selected bond lengths (Å)

Re—O	1.7649 (18)
Re—N1	2.1411 (19)
Re—N2	2.1442 (18)
I1—I2	2.9222 (12)

**Table 2:** Hydrogen-bond geometry (Å, °)

*D*—H⋯*A*	*D*—H	H⋯*A*	*D*⋯*A*	*D*—H⋯*A*
C15—H15⋯O	0.95	2.39	2.914 (3)	114
C25—H25⋯O	0.95	2.38	2.906 (3)	115
C11—H11⋯O^i^	0.95	2.39	2.913 (3)	115
C21—H21⋯O^i^	0.95	2.37	2.908 (3)	115
C22—H22⋯O^ii^	0.95	2.41	3.309 (3)	157

Symmetry codes: (i) 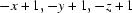; (ii) 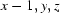.

**Fig. 1.**
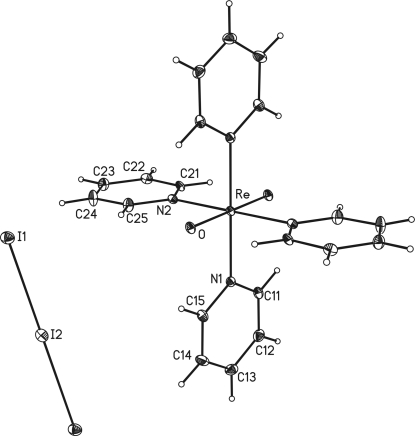
The molecular structure of (I) showing ellipsoids drawn at the 30% probability level. The unlabelled atoms of the cation are generated by the symmetry operation (1–x, 1–y, 1–z) and the unlabelled I atom by (2–x, 2–y, 2–z).

**Fig. 2.**
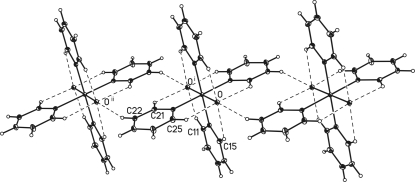
A part of the crystal structure showing formation of C—H···O hydrogen bonding. [symmetry code (i) -x + 1, -y + 1, -z + 1; (ii) x - 1, y, z.]

**Fig. 3.**
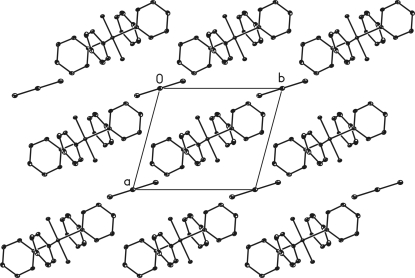
A packing diagram of (I) showing layers of cations and anions. Hydrogen atoms are omitted for clarity.

## supplementary crystallographic information

### Comment

The crystal structure of a salt containing [ReO_2_(C_5_H_5_N)_4_]^+ ^cation was first investigated by Calvo *et al.*, (1971). The authors obtained dioxidotetra(pyridine)rhenium(V) chloride dihydrate in the reaction between trichloridooxidobis(triphenylphosphine)rhenium(V) (Johnson *et al.*, 1967) and hot pyridine used in excess. The crystal structure of [ReO_2_(C_5_H_5_N)_4_]Cl^.^2H_2_O was redetermined by Lock & Turner (1978). The cation [ReO_2_(C_5_H_5_N)_4_]^+^ was also described by Luck & O'Neill (2001) as [ReO_2_(C_5_H_5_N)_4_[OH]^.^1.75H_2_O salt. This salt was prepared by dissolving ReCl(H_2_)(PMePh_2_)_4 _in the mixture of benzene, pyridine, water and hexane.

The crystal structure of *trans*-dioxidotetra(pyridine)rhenium(V) triiodide comprises of [ReO_2_(C_5_H_5_N)_4_]^+ ^cations and I_3_^- ^anions (Fig. 1). Both ions are located on centres of symmetry. The cation is a distorted octahedron, with two *oxido* (terminal) ligands in *trans *arrangement and four pyridine ligands in equatorial positions.

The average Re—O and Re—N bond distances equal 1.765 (2), 2.143 (2) Å, respectively, and are in good agreement with values reported by Calvo *et al*., (1971), Lock & Turner (1978) and Luck & O'Neill (2001). Moreover, comparing the values of O—Re—O angle comparatively small differences between previous and present results can be observed. In the crystal structure reported here this angle equals 180° and reported for other salts is 171 (1)° (Calvo *et al.*, 1971) and 174.5 (4)° (Lock & Turner, 1978). Similarly, the value of N—Re—N*_trans_* angles in [ReO_2_(C_5_H_5_N)_4_]I_3_ equals 180° and the analogous complex cations that have been determined previously have near linear arrangement of the N—Re—N*_trans_* moiety. These angles are 176 (2) and 170 (1)° (Calvo *et al.*, 1971), and 173.9 (4) and 175.2 (6)° (Lock & Turner, 1978). The comparatively weak intramolecular hydrogen bonds such as C—H···O can be observed (Fig. 2, Table 2).

The molecular packing in the crystal structure can be described as layers perpendicular to [100] direction which consist of the complex cations (Fig. 3). The I_3_^-^ anions are located between the layers of [ReO_2_(C_5_H_5_N)_4_]^+^ cations. In the crystal packing there are intermolecular stacking interactions between pyridine rings with centroid-centroid distance of 3.831 (2) Å and a slip angle 25°. These values are comparable with the corresponding values reported for transition-metal pyridine fragments (Janiak, 2000). (The ring centroid contacts range between 3.4 and 3.8 Å and the angle averages 27°).

### Experimental

Rhenium(III) iodide 0.2982 g (0.1753 mmol) was refluxed in dry pyridine (5 ml) (62 mmol) for 3 h at 423 K. The mixture was allowed to evaporate in air at high temperature to give a greenish brown precipitate. The complex was recrystallized from methanol to yield orange blocks of (I).

### Refinement

All hydrogen atoms were placed in calculated positions and refined using riding model [C—H = 0.95 Å and *U*_iso_(H) = 1.2*U*_eq_(C)]. The highest peak and the deepest hole in the final difference map were 1.07 Å from N1 and 0.78 Å from Re, respectively.

### Crystal data

**Table:**

[ReO_2_(C_5_H_5_N)_4_]I_3_	*Z* = 1
*M**_r_* = 915.30	*F*(000) = 418
Triclinic, *P*1	*D*_x_ = 2.370 Mg m^−^^3^
Hall symbol: -P 1	Mo *K*α radiation, λ = 0.71073 Å
*a* = 7.993 (3) Å	Cell parameters from 11676 reflections
*b* = 9.100 (3) Å	θ = 4.5–38.4°
*c* = 9.356 (3) Å	µ = 8.37 mm^−^^1^
α = 92.45 (4)°	*T* = 100 K
β = 102.41 (4)°	Block, orange
γ = 104.10 (4)°	0.10 × 0.10 × 0.07 mm
*V* = 641.3 (4) Å^3^	

### Data collection

**Table:**

Oxford Diffraction Xcalibur PX KM-4-CCD diffractometer	4298 independent reflections
Radiation source: fine-focus sealed tube	3593 reflections with *I* > 2σ(*I*)
graphite	*R*_int_ = 0.027
φ and ω scans	θ_max_ = 32.5°, θ_min_ = 4.5°
Absorption correction: analytical (*CrysAlis RED*; Oxford Diffraction, 2006)	*h* = −12→11
*T*_min_ = 0.411, *T*_max_ = 0.656	*k* = −9→13
11244 measured reflections	*l* = −14→14

### Refinement

**Table:**

Refinement on *F*^2^	Primary atom site location: structure-invariant direct methods
Least-squares matrix: full	Secondary atom site location: difference Fourier map
*R*[*F*^2^ > 2σ(*F*^2^)] = 0.019	Hydrogen site location: inferred from neighbouring sites
*wR*(*F*^2^) = 0.031	H-atom parameters constrained
*S* = 1.04	*w* = 1/[σ^2^(*F*_o_^2^) + (0.008*P*)^2^] where *P* = (*F*_o_^2^ + 2*F*_c_^2^)/3
4298 reflections	(Δ/σ)_max_ = 0.003
139 parameters	Δρ_max_ = 1.40 e Å^−^^3^
0 restraints	Δρ_min_ = −1.08 e Å^−^^3^

### Special details

**Table:**

Geometry. All e.s.d.'s (except the e.s.d. in the dihedral angle between two l.s. planes) are estimated using the full covariance matrix. The cell e.s.d.'s are taken into account individually in the estimation of e.s.d.'s in distances, angles and torsion angles; correlations between e.s.d.'s in cell parameters are only used when they are defined by crystal symmetry. An approximate (isotropic) treatment of cell e.s.d.'s is used for estimating e.s.d.'s involving l.s. planes.
Refinement. Refinement of *F*^2^ against ALL reflections. The weighted *R*-factor *wR* and goodness of fit *S* are based on *F*^2^, conventional *R*-factors *R* are based on *F*, with *F* set to zero for negative *F*^2^. The threshold expression of *F*^2^ > σ(*F*^2^) is used only for calculating *R*-factors(gt) *etc*. and is not relevant to the choice of reflections for refinement. *R*-factors based on *F*^2^ are statistically about twice as large as those based on *F*, and *R*- factors based on ALL data will be even larger.

### Fractional atomic coordinates and isotropic or equivalent isotropic displacement parameters (Å^2^)

**Table:**

	*x*	*y*	*z*	*U*_iso_*/*U*_eq_	
Re	0.5000	0.5000	0.5000	0.01065 (3)	
I1	0.92874 (2)	1.171742 (18)	0.747033 (17)	0.02194 (4)	
I2	1.0000	1.0000	1.0000	0.01924 (5)	
O	0.7122 (2)	0.62887 (16)	0.53698 (16)	0.0144 (3)	
N1	0.5561 (2)	0.4374 (2)	0.71983 (19)	0.0132 (4)	
C11	0.4386 (3)	0.3225 (2)	0.7640 (2)	0.0159 (5)	
H11	0.3318	0.2707	0.6957	0.019*	
C12	0.4708 (3)	0.2798 (3)	0.9045 (3)	0.0213 (5)	
H12	0.3873	0.1988	0.9315	0.026*	
C13	0.6234 (3)	0.3541 (3)	1.0059 (3)	0.0225 (6)	
H13	0.6468	0.3251	1.1031	0.027*	
C14	0.7421 (3)	0.4718 (3)	0.9631 (3)	0.0223 (5)	
H14	0.8480	0.5260	1.0310	0.027*	
C15	0.7045 (3)	0.5096 (3)	0.8203 (2)	0.0176 (5)	
H15	0.7872	0.5903	0.7918	0.021*	
N2	0.3956 (2)	0.6744 (2)	0.58055 (19)	0.0126 (4)	
C21	0.2226 (3)	0.6460 (3)	0.5840 (2)	0.0148 (5)	
H21	0.1481	0.5467	0.5506	0.018*	
C22	0.1494 (3)	0.7552 (3)	0.6340 (2)	0.0173 (5)	
H22	0.0268	0.7312	0.6337	0.021*	
C23	0.2564 (3)	0.8991 (3)	0.6844 (3)	0.0196 (5)	
H23	0.2092	0.9762	0.7192	0.024*	
C24	0.4346 (3)	0.9288 (3)	0.6833 (3)	0.0229 (6)	
H24	0.5119	1.0265	0.7190	0.027*	
C25	0.4992 (3)	0.8152 (2)	0.6299 (3)	0.0189 (5)	
H25	0.6211	0.8374	0.6279	0.023*	

### Atomic displacement parameters (Å^2^)

**Table:**

	*U*^11^	*U*^22^	*U*^33^	*U*^12^	*U*^13^	*U*^23^
Re	0.00779 (7)	0.01136 (7)	0.01158 (6)	0.00084 (5)	0.00184 (5)	−0.00043 (5)
I1	0.01743 (9)	0.02567 (9)	0.02152 (8)	0.00406 (7)	0.00338 (7)	0.00427 (7)
I2	0.01598 (12)	0.02278 (12)	0.01852 (11)	0.00268 (10)	0.00608 (9)	0.00035 (9)
O	0.0099 (8)	0.0144 (8)	0.0166 (8)	0.0008 (7)	0.0020 (7)	−0.0025 (7)
N1	0.0123 (10)	0.0131 (9)	0.0145 (9)	0.0044 (8)	0.0026 (8)	−0.0003 (8)
C11	0.0131 (12)	0.0146 (11)	0.0182 (11)	−0.0002 (10)	0.0043 (10)	−0.0013 (9)
C12	0.0291 (15)	0.0172 (12)	0.0199 (12)	0.0060 (12)	0.0101 (11)	0.0051 (10)
C13	0.0314 (16)	0.0256 (13)	0.0152 (11)	0.0152 (13)	0.0064 (11)	0.0039 (10)
C14	0.0191 (14)	0.0289 (14)	0.0170 (12)	0.0088 (12)	−0.0019 (10)	−0.0021 (11)
C15	0.0141 (12)	0.0178 (12)	0.0181 (11)	0.0005 (10)	0.0029 (10)	−0.0013 (10)
N2	0.0098 (10)	0.0145 (9)	0.0123 (9)	0.0015 (8)	0.0019 (8)	0.0004 (8)
C21	0.0110 (12)	0.0148 (11)	0.0144 (11)	−0.0006 (10)	−0.0009 (9)	−0.0027 (9)
C22	0.0112 (12)	0.0230 (13)	0.0182 (11)	0.0046 (11)	0.0040 (10)	0.0020 (10)
C23	0.0192 (13)	0.0187 (12)	0.0236 (12)	0.0080 (11)	0.0076 (11)	−0.0006 (10)
C24	0.0171 (13)	0.0140 (12)	0.0361 (14)	0.0007 (11)	0.0087 (12)	−0.0061 (11)
C25	0.0127 (12)	0.0163 (12)	0.0268 (13)	0.0003 (10)	0.0067 (10)	−0.0006 (10)

### Geometric parameters (Å, °)

**Table:**

Re—O^i^	1.7649 (18)	C13—H13	0.9500
Re—O	1.7649 (18)	C14—C15	1.382 (3)
Re—N1^i^	2.1411 (19)	C14—H14	0.9500
Re—N1	2.1411 (19)	C15—H15	0.9500
Re—N2^i^	2.1442 (18)	N2—C25	1.344 (3)
Re—N2	2.1442 (18)	N2—C21	1.351 (3)
I1—I2	2.9222 (12)	C21—C22	1.382 (3)
I2—I1^ii^	2.9222 (12)	C21—H21	0.9500
N1—C15	1.346 (3)	C22—C23	1.378 (3)
N1—C11	1.368 (3)	C22—H22	0.9500
C11—C12	1.375 (3)	C23—C24	1.386 (3)
C11—H11	0.9500	C23—H23	0.9500
C12—C13	1.375 (4)	C24—C25	1.383 (3)
C12—H12	0.9500	C24—H24	0.9500
C13—C14	1.383 (3)	C25—H25	0.9500
			
O^i^—Re—O	180.0	C12—C13—H13	120.8
O^i^—Re—N1^i^	89.50 (8)	C14—C13—H13	120.8
O—Re—N1^i^	90.50 (8)	C15—C14—C13	119.2 (3)
O^i^—Re—N1	90.50 (8)	C15—C14—H14	120.4
O—Re—N1	89.50 (8)	C13—C14—H14	120.4
N1^i^—Re—N1	180.0	N1—C15—C14	123.0 (2)
O^i^—Re—N2^i^	89.76 (7)	N1—C15—H15	118.5
O—Re—N2^i^	90.24 (7)	C14—C15—H15	118.5
N1^i^—Re—N2^i^	88.04 (7)	C25—N2—C21	117.65 (18)
N1—Re—N2^i^	91.96 (7)	C25—N2—Re	121.51 (15)
O^i^—Re—N2	90.24 (7)	C21—N2—Re	120.85 (15)
O—Re—N2	89.76 (7)	N2—C21—C22	122.8 (2)
N1^i^—Re—N2	91.96 (7)	N2—C21—H21	118.6
N1—Re—N2	88.04 (7)	C22—C21—H21	118.6
N2^i^—Re—N2	180.0	C23—C22—C21	119.2 (2)
I1—I2—I1^ii^	180.0	C23—C22—H22	120.4
C15—N1—C11	117.36 (19)	C21—C22—H22	120.4
C15—N1—Re	122.15 (15)	C22—C23—C24	118.4 (2)
C11—N1—Re	120.48 (16)	C22—C23—H23	120.8
N1—C11—C12	121.8 (2)	C24—C23—H23	120.8
N1—C11—H11	119.1	C25—C24—C23	119.5 (2)
C12—C11—H11	119.1	C25—C24—H24	120.2
C13—C12—C11	120.3 (2)	C23—C24—H24	120.2
C13—C12—H12	119.9	N2—C25—C24	122.4 (2)
C11—C12—H12	119.9	N2—C25—H25	118.8
C12—C13—C14	118.5 (2)	C24—C25—H25	118.8
			
O^i^—Re—N1—C15	−173.72 (16)	O^i^—Re—N2—C25	−179.56 (17)
O—Re—N1—C15	6.28 (16)	O—Re—N2—C25	0.44 (17)
N2^i^—Re—N1—C15	96.50 (16)	N1^i^—Re—N2—C25	−90.05 (18)
N2—Re—N1—C15	−83.50 (16)	N1—Re—N2—C25	89.95 (18)
O^i^—Re—N1—C11	5.08 (14)	O^i^—Re—N2—C21	0.76 (16)
O—Re—N1—C11	−174.92 (14)	O—Re—N2—C21	−179.24 (16)
N2^i^—Re—N1—C11	−84.70 (15)	N1^i^—Re—N2—C21	90.27 (17)
N2—Re—N1—C11	95.30 (15)	N1—Re—N2—C21	−89.73 (17)
C15—N1—C11—C12	−1.0 (3)	C25—N2—C21—C22	0.8 (3)
Re—N1—C11—C12	−179.90 (15)	Re—N2—C21—C22	−179.55 (16)
N1—C11—C12—C13	0.7 (3)	N2—C21—C22—C23	−0.8 (3)
C11—C12—C13—C14	0.2 (3)	C21—C22—C23—C24	−0.2 (3)
C12—C13—C14—C15	−0.7 (3)	C22—C23—C24—C25	1.1 (4)
C11—N1—C15—C14	0.5 (3)	C21—N2—C25—C24	0.2 (3)
Re—N1—C15—C14	179.34 (16)	Re—N2—C25—C24	−179.47 (18)
C13—C14—C15—N1	0.4 (3)	C23—C24—C25—N2	−1.1 (4)

Symmetry codes: (i) −*x*+1, −*y*+1, −*z*+1; (ii) −*x*+2, −*y*+2, −*z*+2.

### Hydrogen-bond geometry (Å, °)

**Table:**

*D*—H···*A*	*D*—H	H···*A*	*D*···*A*	*D*—H···*A*
C15—H15···O	0.95	2.39	2.914 (3)	114
C25—H25···O	0.95	2.38	2.906 (3)	115
C11—H11···O^i^	0.95	2.39	2.913 (3)	115
C21—H21···O^i^	0.95	2.37	2.908 (3)	115
C22—H22···O^iii^	0.95	2.41	3.309 (3)	157

Symmetry codes: (i) −*x*+1, −*y*+1, −*z*+1; (iii) *x*−1, *y*, *z*.
